# Supplementary material for: Genetic variability and structure of the Olive Field Mouse: a sigmodontine rodent in a biodiversity hotspot of southern Chile
Source: PeerJ. 2019 May 21;7:e6955. doi: 10.7717/peerj.6955 (PMC6534109; doi:10.7717/peerj.6955)
Supplement: Table S1 — List of specimens analyzed and geographic coordinates, with also the genetic marker used (Microsatellite STR and/or mitochondrial) [file peerj-07-6955-s001.docx]

Table S1. Localities sampled and geographic coordinates

| Collection number | Geopolitical division (Region, Province, Locality) | SP | Latitude | Longitude | STR | Mitochondrial |
| --- | --- | --- | --- | --- | --- | --- |
| NK105843 | Región de Los Rios, Panguipulli | 1 | -39.7246667 | -72.3981111 | x |  |
| NK105848 | Región de Los Rios, Panguipulli | 1 | -39.7246667 | -72.3981111 | x |  |
| NK105858 | Región de Los Rios, Panguipulli | 1 | -39.7246667 | -72.3981111 | x |  |
| NK160715 | Región de Los Rios, Panguipulli | 2 | -39.49091667 | -72.53902778 | x |  |
| NK104807 | Región de Los Rios, Panguipulli | 3 | -39.73665 | -72.22749 | x |  |
| NK104808 | Región de Los Rios, Panguipulli | 3 | -39.73665 | -72.22749 | x |  |
| NK104810 | Región de Los Rios, Panguipulli | 3 | -39.73665 | -72.22749 | x | x |
| NK104811 | Región de Los Rios, Panguipulli | 3 | -39.73665 | -72.22749 | x | x |
| NK129001 | Región de Los Rios, Panguipulli | 4 | -39.4893 | -72.5582 | x |  |
| UCK222 | Región de Los Rios, Panguipulli | 5 | -39.63864124 | -72.22079868 | x |  |
| UCK225 | Región de Los Rios, Panguipulli | 5 | -39.63864124 | -72.22079868 | x |  |
| UCK227 | Región de Los Rios, Panguipulli | 5 | -39.63864124 | -72.22079868 | x |  |
| UCK232 | Región de Los Rios, Panguipulli | 5 | -39.63864124 | -72.22079868 | x |  |
| UCK233 | Región de Los Rios, Panguipulli | 5 | -39.63864124 | -72.22079868 | x |  |
| UCK231 | Región de Los Rios, Panguipulli | 5 | -39.63864124 | -72.22079868 | x |  |
| UCK76 | Región de Los Rios, Valdivia | 1 | -39.69809571 | -73.37987548 | x |  |
| UCK87 | Región de Los Rios, Valdivia | 1 | -39.69809571 | -73.37987548 | x |  |
| UCK94 | Región de Los Rios, Valdivia | 1 | -39.69809571 | -73.37987548 | x |  |
| NK108767 | Región de Los Rios, Valdivia | 2 | -39.806 | -73.0688 | x | x |
| NK108766 | Región de Los Rios, Valdivia | 3 | -39.8074 | -73.1538 | x | x |
| NK108765 | Región de Los Rios, Valdivia | 3 | -39.8074 | -73.1538 | x | x |
| NK108764 | Región de Los Rios, Valdivia | 3 | -39.8074 | -73.1538 | x |  |
| NK108763 | Región de Los Rios, Valdivia | 3 | -39.8074 | -73.1538 | x | x |
| NK108762 | Región de Los Rios, Valdivia | 2 | -39.806 | -73.0688 | x | x |
| NK108760 | Región de Los Rios, Valdivia | 2 | -39.806 | -73.0688 | x | x |
| NK108759 | Región de Los Rios, Valdivia | 2 | -39.806 | -73.0688 | x | x |
| NK108755 | Región de Los Rios, Valdivia | 3 | -39.8074 | -73.1538 | x | x |
| NK108754 | Región de Los Rios, Valdivia | 3 | -39.8074 | -73.1538 | x | x |
| NK108753 | Región de Los Rios, Valdivia | 3 | -39.8074 | -73.1538 | x |  |
| NK108752 | Región de Los Rios, Valdivia | 3 | -39.8074 | -73.1538 | x |  |
| NK108751 | Región de Los Rios, Valdivia | 3 | -39.8074 | -73.1538 | x | x |
| NK108750 | Región de Los Rios, Valdivia | 3 | -39.8074 | -73.1538 | x | x |
| NK108745 | Región de Los Rios, Valdivia | 3 | -39.8074 | -73.1538 | x | x |
| NK108744 | Región de Los Rios, Valdivia | 3 | -39.8074 | -73.1538 | x | x |
| NK108743 | Región de Los Rios, Valdivia | 3 | -39.8074 | -73.1538 | x | x |
| NK108742 | Región de Los Rios, Valdivia | 3 | -39.8074 | -73.1538 | x | x |
| NK108741 | Región de Los Rios, Valdivia | 3 | -39.8074 | -73.1538 | x | x |
| NK108740 | Región de Los Rios, Valdivia | 3 | -39.8074 | -73.1538 | x | x |
| NK106274 | Región de Los Rios, Valdivia | 3 | -39.8074 | -73.1538 | x |  |
| NK106265 | Región de Los Rios, Valdivia | 3 | -39.8074 | -73.1538 | x | x |
| NK106264 | Región de Los Rios, Valdivia | 3 | -39.8074 | -73.1538 | x |  |
| NK109490 | Región de Los Lagos, La Picada | 1 | -41.01715 | -72.5504 | x | x |
| NK109488 | Región de Los Lagos, Bahia Mansa | 1 | -40.58195 | -73.6999 | x |  |
| NK109483 | Región de Los Lagos, La Picada | 1 | -41.01715 | -72.5504 | x | x |
| NK109482 | Región de Los Lagos, La Picada | 1 | -41.01715 | -72.5504 | x | x |
| NK109079 | Región de Los Lagos, Bahia Mansa | 1 | -40.58195 | -73.6999 | x |  |
| NK109077 | Región de Los Lagos, Bahia Mansa | 1 | -40.58195 | -73.6999 | x | x |
| NK109073 | Región de Los Lagos, Bahia Mansa | 1 | -40.58195 | -73.6999 | x |  |
| NK109071 | Región de Los Lagos, Bahia Mansa | 1 | -40.58195 | -73.6999 | x | x |
| NK109047 | Región de Los Lagos, Bahia Mansa | 1 | -40.58195 | -73.6999 | x |  |
| NK109081 | Región de Los Lagos, Bahia Mansa | 1 | -40.58195 | -73.6999 |  | x |
| NK109083 | Región de Los Lagos, Bahia Mansa | 1 | -40.58195 | -73.6999 | x |  |
| NK109084 | Región de Los Lagos, Bahia Mansa | 1 | -40.58195 | -73.6999 | x |  |
| NK109086 | Región de Los Lagos, Bahia Mansa | 1 | -40.58195 | -73.6999 | x | x |
| NK109087 | Región de Los Lagos, Bahia Mansa | 1 | -40.58195 | -73.6999 |  | x |
| NK109088 | Región de Los Lagos, Bahia Mansa | 1 | -40.58195 | -73.6999 | x |  |
| NK109089 | Región de Los Lagos, Bahia Mansa | 1 | -40.58195 | -73.6999 | x | x |
| NK109091 | Región de Los Lagos, Bahia Mansa | 1 | -40.58195 | -73.6999 | x |  |
| NK105169 | Región de Los Lagos, Las Quemas | 1 | -41.416 | -73.191 | x | x |
| NK105164 | Región de Los Lagos, Las Quemas | 1 | -41.416 | -73.191 | x | x |
| NK105163 | Región de Los Lagos, Las Quemas | 1 | -41.416 | -73.191 | x | x |
| NK105162 | Región de Los Lagos, Las Quemas | 1 | -41.416 | -73.191 | x | x |
| NK105161 | Región de Los Lagos, Las Quemas | 1 | -41.416 | -73.191 | x | x |
| NK105160 | Región de Los Lagos, Las Quemas | 1 | -41.416 | -73.191 | x | x |
| NK105159 | Región de Los Lagos, Las Quemas | 1 | -41.416 | -73.191 | x | x |
| NK105158 | Región de Los Lagos, Las Quemas | 1 | -41.416 | -73.191 | x | x |
| NK105156 | Región de Los Lagos, Las Quemas | 1 | -41.416 | -73.191 | x | x |
| NK105155 | Región de Los Lagos, Las Quemas | 1 | -41.416 | -73.191 | x | x |
| NK95661 | Región de Los Lagos, Chiloe | 1 | -41.88316667 | -73.67211111 | x | x |
| NK95657 | Región de Los Lagos, Chiloe | 1 | -41.88316667 | -73.67211111 | x | x |
| NK95655 | Región de Los Lagos, Chiloe | 1 | -41.88316667 | -73.67211111 | x | x |
| NK95654 | Región de Los Lagos, Chiloe | 1 | -41.88316667 | -73.67211111 | x |  |
| NK95653 | Región de Los Lagos, Chiloe | 1 | -41.88316667 | -73.67211111 | x | x |
| NK95651 | Región de Los Lagos, Chiloe | 1 | -41.88316667 | -73.67211111 | x | x |
| NK95650 | Región de Los Lagos, Chiloe | 1 | -41.88316667 | -73.67211111 | x | x |
| NK95649 | Región de Los Lagos, Chiloe | 1 | -41.88316667 | -73.67211111 | x | x |
| NK95647 | Región de Los Lagos, Chiloe | 1 | -41.88316667 | -73.67211111 | x | x |
| NK95643 | Región de Los Lagos, Chiloe | 1 | -41.88316667 | -73.67211111 | x | x |
| NK95642 | Región de Los Lagos, Chiloe | 1 | -41.88316667 | -73.67211111 | x | x |
| NK95641 | Región de Los Lagos, Chiloe | 1 | -41.88316667 | -73.67211111 | x | x |
| UCK926 | Región de Los Lagos, Caulin | 2 | -41.83839431 | -73.60588788 | x |  |
| UCK925 | Región de Los Lagos, Caulin | 2 | -41.83839431 | -73.60588788 | x |  |
| UCK924 | Región de Los Lagos, Caulin | 2 | -41.83839431 | -73.60588788 | x |  |
| UCK923 | Región de Los Lagos, Caulin | 2 | -41.83839431 | -73.60588788 | x |  |
| UCK922 | Región de Los Lagos, Caulin | 2 | -41.83839431 | -73.60588788 | x |  |
| UCK917 | Región de Los Lagos, Caulin | 2 | -41.83839431 | -73.60588788 | x |  |
| UCK913 | Región de Los Lagos, Caulin | 2 | -41.83839431 | -73.60588788 | x | x |
| UCK910 | Región de Los Lagos, Caulin | 2 | -41.83839431 | -73.60588788 | x |  |
| UCK908 | Región de Los Lagos, Caulin | 2 | -41.83839431 | -73.60588788 | x |  |
| UCK907 | Región de Los Lagos, Caulin | 2 | -41.83839431 | -73.60588788 | x |  |
| UCK921 | Región de Los Lagos, SD3 | 3 | -41.88226454 | -73.67659663 | x |  |
| UCK920 | Región de Los Lagos, SD3 | 3 | -41.88226454 | -73.67659663 | x |  |
| UCK919 | Región de Los Lagos, SD3 | 3 | -41.88226454 | -73.67659663 | x |  |
| UCK918 | Región de Los Lagos, SD3 | 3 | -41.88226454 | -73.67659663 | x | x |
| UCK916 | Región de Los Lagos, SD3 | 3 | -41.88226454 | -73.67659663 | x |  |
| UCK915 | Región de Los Lagos, SD3 | 3 | -41.88226454 | -73.67659663 | x |  |
| UCK914 | Región de Los Lagos, SD3 | 3 | -41.88226454 | -73.67659663 | x |  |
| UCK912 | Región de Los Lagos, SD3 | 3 | -41.88226454 | -73.67659663 | x |  |
| UCK911 | Región de Los Lagos, SD3 | 3 | -41.88226454 | -73.67659663 | x |  |
| UCK909 | Región de Los Lagos, SD3 | 3 | -41.88226454 | -73.67659663 | x |  |
| NK105464 | Región de Los Lagos, SD2 | 4 | -41.88381 | -73.66653 | x | x |
| NK105463 | Región de Los Lagos, SD2 | 4 | -41.88381 | -73.66653 | x | x |
| NK105462 | Región de Los Lagos, SD2 | 4 | -41.88381 | -73.66653 | x | x |
| NK105460 | Región de Los Lagos, SD2 | 4 | -41.88381 | -73.66653 | x | x |
| NK105459 | Región de Los Lagos, SD2 | 4 | -41.88381 | -73.66653 | x | x |
| NK105458 | Región de Los Lagos, SD2 | 4 | -41.88381 | -73.66653 | x |  |
| NK105456 | Región de Los Lagos, SD2 | 4 | -41.88381 | -73.66653 | x |  |
| NK105454 | Región de Los Lagos, SD2 | 4 | -41.88381 | -73.66653 | x | x |
| NK105453 | Región de Los Lagos, SD2 | 4 | -41.88381 | -73.66653 | x |  |
| NK105451 | Región de Los Lagos, SD2 | 4 | -41.88381 | -73.66653 | x | x |
| NK105450 | Región de Los Lagos, SD2 | 4 | -41.88381 | -73.66653 | x |  |
| NK105448 | Región de Los Lagos, SD2 | 4 | -41.88381 | -73.66653 | x | x |
| NK105444 | Región de Los Lagos, SD2 | 4 | -41.88381 | -73.66653 | x | x |
| NK160539 | Región de Los Lagos, SD | 5 | -41.86847222 | -73.68125 | x | x |
| NK160536 | Región de Los Lagos, SD | 5 | -41.86847222 | -73.68125 | x | x |
| NK160535 | Región de Los Lagos, SD | 5 | -41.86847222 | -73.68125 | x | x |
| NK160534 | Región de Los Lagos, SD | 5 | -41.86847222 | -73.68125 | x | x |
| UCK672 | Región de Los Lagos, Cucao | 1 | -42.621667 | -74.105833 | x |  |
| UCK674 | Región de Los Lagos, Cucao | 1 | -42.621667 | -74.105833 | x | x |
| UCK675 | Región de Los Lagos, Cucao | 1 | -42.621667 | -74.105833 | x | x |
| UCK676 | Región de Los Lagos, Cucao | 1 | -42.621667 | -74.105833 | x | x |
| UCK680 | Región de Los Lagos, Cucao | 1 | -42.621667 | -74.105833 | x | x |
| UCK683 | Región de Los Lagos, Cucao | 1 | -42.621667 | -74.105833 | x | x |
| UCK686 | Región de Los Lagos, Cucao | 1 | -42.621667 | -74.105833 | x |  |
| UCK687 | Región de Los Lagos, Cucao | 1 | -42.621667 | -74.105833 | x | x |
| UCK688 | Región de Los Lagos, Cucao | 1 | -42.621667 | -74.105833 | x | x |
| UCK689 | Región de Los Lagos, Cucao | 1 | -42.621667 | -74.105833 | x |  |
| UCK691 | Región de Los Lagos, Cucao | 1 | -42.621667 | -74.105833 | x | x |
| UCK692 | Región de Los Lagos, Cucao | 1 | -42.621667 | -74.105833 | x | x |
| UCK693 | Región de Los Lagos, Cucao | 1 | -42.621667 | -74.105833 | x | x |
| UCK694 | Región de Los Lagos, Cucao | 1 | -42.621667 | -74.105833 | x | x |
| UCK695 | Región de Los Lagos, Cucao | 1 | -42.621667 | -74.105833 | x | x |
| UCK696 | Región de Los Lagos, Cucao | 1 | -42.621667 | -74.105833 | x |  |
| UCK698 | Región de Los Lagos, Cucao | 1 | -42.621667 | -74.105833 | x |  |
| UCK699 | Región de Los Lagos, Cucao | 1 | -42.621667 | -74.105833 | x | x |
| UCK700 | Región de Los Lagos, Cucao | 1 | -42.621667 | -74.105833 | x | x |
| UCK701 | Región de Los Lagos, Cucao | 1 | -42.621667 | -74.105833 | x | x |
| UCK702 | Región de Los Lagos, Cucao | 1 | -42.621667 | -74.105833 | x | x |
| UCK703 | Región de Los Lagos, Cucao | 1 | -42.621667 | -74.105833 | x | x |
| UCK705 | Región de Los Lagos, Cucao | 1 | -42.621667 | -74.105833 | x | x |
| UCK706 | Región de Los Lagos, Cucao | 1 | -42.621667 | -74.105833 | x | x |
| UCK710 | Región de Los Lagos, Cucao | 1 | -42.621667 | -74.105833 | x | x |
| UCK711 | Región de Los Lagos, Cucao | 1 | -42.621667 | -74.105833 | x | x |
| UCK712 | Región de Los Lagos, Cucao | 1 | -42.621667 | -74.105833 | x | x |
| NK129185 | Región de Los Lagos, Palena | 1 | -43.626133 | -71.7534 | x |  |
| NK129184 | Región de Los Lagos, Palena | 1 | -43.626133 | -71.7534 | x |  |
| NK129183 | Región de Los Lagos, Palena | 2 | -43.620366 | -71.775266 | x |  |
| NK129150 | Región de Los Lagos, Palena | 2 | -43.620366 | -71.775266 | x |  |
| NK129152 | Región de Los Lagos, Palena | 2 | -43.620366 | -71.775266 | x |  |
| NK129157 | Región de Los Lagos, Palena | 2 | -43.620366 | -71.775266 | x |  |
| NK129158 | Región de Los Lagos, Palena | 2 | -43.620366 | -71.775266 | x |  |
| NK129148 | Región de Los Lagos, Palena | 2 | -43.620366 | -71.775266 | x |  |
| NK129130 | Región de Los Lagos, Santa Lucia | 1 | -43.617716 | -72.334433 | x |  |
| NK129144 | Región de Los Lagos, Santa Lucia | 1 | -43.617716 | -72.334433 | x |  |
| NK129134 | Región de Los Lagos, Santa Lucia | 1 | -43.617716 | -72.334433 | x |  |
| NK129143 | Región de Los Lagos, Santa Lucia | 1 | -43.617716 | -72.334433 | x |  |
| NK105410 | Región de Aysen, Rio Simpson | 1 | -45.4621 | -72.322 | x |  |
| NK105413 | Región de Aysen, Rio Simpson | 1 | -45.4621 | -72.322 | x |  |
| NK105411 | Región de Aysen, Rio Simpson | 1 | -45.4621 | -72.322 | x |  |
| NK105415 | Región de Aysen, Rio Simpson | 2 | -45.459 | -72.347 | x |  |
| NK96551 | Región de Aysen, Rio Simpson | 3 | -45.46163889 | -72.32483333 | x |  |
| NK96564 | Región de Aysen, Rio Simpson | 3 | -45.46163889 | -72.32483333 | x |  |
| NK96567 | Región de Aysen, Rio Simpson | 3 | -45.46163889 | -72.32483333 | x |  |
| NK96553 | Región de Aysen, Rio Simpson | 3 | -45.46163889 | -72.32483333 | x |  |
| NK96563 | Región de Aysen, Rio Simpson | 3 | -45.46163889 | -72.32483333 | x |  |
| NK95256 | Región de Aysen, Mininco | 1 | -45.51743333 | -71.86371667 | x |  |
| NK95254 | Región de Aysen, Mininco | 1 | -45.51743333 | -71.86371667 | x |  |
| NK95253 | Región de Aysen, Mininco | 1 | -45.51743333 | -71.86371667 | x |  |
| NK95276 | Región de Aysen, Mininco | 1 | -45.51743333 | -71.86371667 | x |  |
| NK95221 | Región de Aysen, Mininco | 1 | -45.51743333 | -71.86371667 | x |  |
| NK95279 | Región de Aysen, Mininco | 1 | -45.51743333 | -71.86371667 | x |  |
| NK95239 | Región de Aysen, Mininco | 1 | -45.51743333 | -71.86371667 | x |  |
| NK95236 | Región de Aysen, Mininco | 1 | -45.51743333 | -71.86371667 | x |  |
| NK95229 | Región de Aysen, Mininco | 1 | -45.51743333 | -71.86371667 | x |  |
| NK95929 | Región de Aysen, Alto Rio Ibañez | 1 | -46.08583333 | -72.581 | x | x |
| NK95937 | Región de Aysen, Alto Rio Ibañez | 1 | -46.08583333 | -72.581 | x |  |
| NK95942 | Región de Aysen, Alto Rio Ibañez | 1 | -46.08583333 | -72.581 | x |  |
| NK95945 | Región de Aysen, Alto Rio Ibañez | 1 | -46.08583333 | -72.581 | x | x |
| NK95949 | Región de Aysen, Alto Rio Ibañez | 1 | -46.08583333 | -72.581 | x | x |
| NK95950 | Región de Aysen, Alto Rio Ibañez | 1 | -46.08583333 | -72.581 | x | x |
| NK95951 | Región de Aysen, Alto Rio Ibañez | 1 | -46.08583333 | -72.581 | x |  |
| NK95954 | Región de Aysen, Alto Rio Ibañez | 1 | -46.08583333 | -72.581 | x |  |
| NK95955 | Región de Aysen, Alto Rio Ibañez | 1 | -46.08583333 | -72.581 | x | x |
| NK95956 | Región de Aysen, Alto Rio Ibañez | 1 | -46.08583333 | -72.581 | x | x |
| NK95960 | Región de Aysen, Alto Rio Ibañez | 1 | -46.08583333 | -72.581 | x | x |
| NK95961 | Región de Aysen, Alto Rio Ibañez | 1 | -46.08583333 | -72.581 | x | x |
| NK95935 | Región de Aysen, Alto Rio Ibañez | 1 | -46.08583333 | -72.581 | x |  |
| NK95948 | Región de Aysen, Alto Rio Ibañez | 1 | -46.08583333 | -72.581 | x |  |
| NK95952 | Región de Aysen, Alto Rio Ibañez | 1 | -46.08583333 | -72.581 | x |  |
| NK95959 | Región de Aysen, Alto Rio Ibañez | 1 | -46.08583333 | -72.581 | x |  |
| NK95941 | Región de Aysen, Rio Ibañez | 1 | -46.08583333 | -72.581 |  | x |
| NK95944 | Región de Ayesn, Rio Ibañez | 1 | -46.08583333 | -72.581 |  | x |
| NK120316 | Región de Aysen, Monumento Nacional Dos Lagunas | 1 | -41.86666667 | -73.66666667 |  | x |
| NK120338 | Región de Aysen, Monumento Nacional Dos Lagunas | 1 | -41.86666667 | -73.66666667 |  | x |
| NK120501 | Región de Aysen, Monumento Nacional Dos Lagunas | 1 | -41.86666667 | -73.66666667 |  | x |
| NK120562 | Región de Aysen, Monumento Nacional Dos Lagunas | 1 | -41.86666667 | -73.66666667 |  | x |
| NK120796 | Región de Aysen, Monumento Nacional Dos Lagunas | 1 | -41.86666667 | -73.66666667 |  | x |
| NK120824 | Región de Aysen, Monumento Nacional Dos Lagunas | 1 | -41.86666667 | -73.66666667 |  | x |
| NK120839 | Región de Aysen, Monumento Nacional Dos Lagunas | 1 | -41.86666667 | -73.66666667 |  | x |
| NK120850 | Región de Aysen, Monumento Nacional Dos Lagunas | 1 | -41.86666667 | -73.66666667 |  | x |
| NK120875 | Región de Aysen, Monumento Nacional Dos Lagunas | 1 | -41.86666667 | -73.66666667 |  | x |
| NK120954 | Región de Aysen, Monumento Nacional Dos Lagunas | 1 | -41.86666667 | -73.66666667 |  | x |
