# Supplementary material for: Genetic variability and structure of the Olive Field Mouse: a sigmodontine rodent in a biodiversity hotspot of southern Chile
Source: PeerJ. 2019 May 21;7:e6955. doi: 10.7717/peerj.6955 (PMC6534109; doi:10.7717/peerj.6955)
Supplement: Table S2 [file peerj-07-6955-s002.docx]

Table S2. Genbank accession for microsatellite primer set

| Locus | Repeat motif | Primer sequences (5´-3´) | Accession |
| --- | --- | --- | --- |
| Aol12 | (gt)10 | F: AAAGCAGCCAGGCATATACA | Pr032816309 |
|  |  | R: CCAGCTCTAAACCCTGTGAG |  |
| Aol28 | (gt)24 | F: GGACGCGCACAGAGACCTAT | Pr032816310 |
|  |  | R: TTTCCAGAATGGATCCCTCA |  |
| Aol44 | (tg)13 | F: GGAGCTCACAAGCAGTCTTTC | Pr032816311 |
|  |  | R: TGAAGGAAAGCCAGAACTGA |  |
| Aol45 | (gt)22 | F: GTACTCCCCTCCCAGGCTAC | Pr032816312 |
|  |  | R: AAACATTCCAGCCACTTTGC |  |
| Aol47 | (tg)14 | F: GGCCAGCTCTGACTGTAGGT | Pr032816313 |
|  |  | R: ACTTCTGGCCTTCACATGCT |  |
| Aol52B | (gt)19 | F: GGCTAAGACTAAGGGGCATT | Pr032816314 |
|  |  | R: GCACTAACTTTCCAATAGAGATG |  |
| Aol53 | (ac)7 | F: CCAAAAGCCGATGCCAGAGT | Pr032816315 |
|  |  | GTACATCTGGAGGGAGGGGT |  |
| Aol55B | (ac)18 | F: AAGGGAGGCAGGGGGTAGAG | Pr032816316 |
|  |  | R: TGTTCTCCTTCTCCCGGTAA |  |
| Aol56B | (ca)10 | F: GGGGCAGCTCTAAACTGAGG | Pr032816317 |
|  |  | R: AGCGGTGCTGGTGTGAAAG |  |
| Aol58 | (ac)25 | F: TGAAAAAGAACACTGCTTGTG | Pr032816318 |
|  |  | R: CTTTGTGCCTCCTGGTAACT |  |
| Aol75 | (gt)26 | F: CAGAATCAGGAATGCCTTAAGGA | Pr032816319 |
|  |  | R: GACTTCTGCACCCAAGGTTTC |  |
| Aol91 | (gt)26 | F: AGGCATGAGGGAACTGAAGA | Pr032816320 |
|  |  | R: CCCCATTCCCTGTATTCTCA |  |
