# Supplementary material for: Genetic variability and structure of the Olive Field Mouse: a sigmodontine rodent in a biodiversity hotspot of southern Chile
Source: PeerJ. 2019 May 21;7:e6955. doi: 10.7717/peerj.6955 (PMC6534109; doi:10.7717/peerj.6955)
Supplement: Table S3 — Pairwise comparison between population above diagonal and p values below diagonal. ( ∗ = p > 0.01) [file peerj-07-6955-s003.docx]

Table 2.

|  | *Panguipulli* | *Valdivia* | *BH_M* | *La_Picada* | *Las_Quemas* | *Chiloe* | *Cucao* | *Palena_Lucia* | *Rio_Simpson* | *Mininco* | *Alto_Rio_Ibañez* |
| --- | --- | --- | --- | --- | --- | --- | --- | --- | --- | --- | --- |
| *Panguipulli* | -- | 0.022 | 0.022 | 0.032 | 0.045 | 0.061 | 0.013 | 0.058 | 0.034 | 0.033 | 0.057 |
| *Valdivia* | 0.000 | -- | 0.025 | 0.016 | 0.026 | 0.031 | 0.046 | 0.083 | 0.059 | 0.061 | 0.081 |
| *BH M* | 0.008 | 0.001 | -- | 0.01 | 0.039 | 0.036 | 0.064 | 0.098 | 0.076 | 0.089 | 0.091 |
| *La Picada* | 0.018* | 0.123* | 0.32* | -- | 0.011 | 0.049 | 0.072 | 0.116 | 0.073 | 0.093 | 0.105 |
| *Las Quemas* | 0.000 | 0.000 | 0.009 | 0.261* | -- | 0.05 | 0.068 | 0.086 | 0.058 | 0.083 | 0.071 |
| *Chiloe* | 0.000 | 0.000 | 0.000 | 0.006 | 0.000 | -- | 0.073 | 0.117 | 0.089 | 0.099 | 0.11 |
| *Cucao* | 0.016* | 0.000 | 0.000 | 0.004 | 0.000 | 0.000 | -- | 0.068 | 0.05 | 0.043 | 0.056 |
| *Palena Lucia* | 0.000 | 0.000 | 0.000 | 0.001 | 0.000 | 0.000 | 0.000 | -- | 0.03 | 0.025 | 0.039 |
| *Rio Simpson* | 0.000 | 0.000 | 0.000 | 0.007 | 0.000 | 0.000 | 0.000 | 0.005 | -- | -0.006 | 0.019 |
| *Mininco* | 0.001 | 0.000 | 0.000 | 0.003 | 0.000 | 0.000 | 0.000 | 0.022* | 0.699* | -- | 0.037 |
| *Alto Rio Ibañez* | 0.000 | 0.000 | 0.000 | 0.000 | 0.000 | 0.000 | 0.000 | 0.000 | 0.005 | 0.002 | -- |
